# Acute Psychosis as Presenting Symptoms of Vitamin B12 Deficiency: A Case Series

**DOI:** 10.7759/cureus.74844

**Published:** 2024-11-30

**Authors:** Manoj Sahoo, Dyuthy R, Anupama Sahu

**Affiliations:** 1 Psychiatry, Tata Main Hospital, Jamshedpur, IND; 2 Psychiatry, Manipal Tata Medical College, Manipal Academy of Higher Education (MAHE), Jamshedpur, IND; 3 Internal Medicine, Tata Main Hospital, Jamshedpur, IND

**Keywords:** acute psychosis, psychiatric symptoms, reversible causes, vegetarian diet, vitamin b12 deficiency

## Abstract

Background: Vitamin B12 deficiency is a prevalent condition affecting a significant proportion of India's population, with implications for mental health. Despite its established link to psychiatric symptoms, vitamin B12 deficiency often remains underdiagnosed. The aim of this study is to highlight the association between vitamin B12 deficiency and acute psychosis, emphasizing the importance of early detection and treatment.

Methods: This is a retrospective case series with inclusion criteria of diagnosis of Acute and Transient Psychotic Disorder (ATPD) and associated vitamin B12 Deficiency over the past five years. Patients with comorbid psychiatric illnesses and substance use other than nicotine, hematological and neurological symptoms associated with vitamin B12 deficiency and significant life stressors prior to onset of illness were excluded from the study. A review of electronic medical records of all these patients was done. Out of these patients, eight cases satisfied the study selection criteria.

Results: Sociodemographic, investigation, and treatment details of all eight patients are presented. All of them received low-dose antipsychotics and vitamin B12 replacement therapy, resulting in significant improvement. Vegetarian dietary habits were identified as a common risk factor.

Conclusion: Vitamin B12 deficiency can manifest solely as ATPD, particularly in individuals following vegetarian diets. Early detection and treatment are crucial to prevent irreversible psychiatric and neurological damage. Routine vitamin B12 level assessments are recommended in patients with mental and psychiatric disturbances.

## Introduction

For decades, vitamin B12 deficiency has been a hidden culprit behind psychiatric symptoms, striking up to 67% of India's population [1]. A significant proportion (33.3%) of indoor psychiatric patients are suffering from vitamin B12 deficiency [2]. Vitamin B12 deficiency has been conclusively linked to a broad spectrum of psychiatric, hematological, and neurological symptoms. Historical reports and contemporary research converge on consistent symptom profiles despite evolving diagnostic specificity. Prominently documented conditions include cognitive impairment (slow cerebration, confusion, memory changes), psychotic episodes (delirium, hallucinations, delusions), mood disorders (depression, reversible manic states), and psychotic spectrum disorders (schizophreniform, acute psychotic states) [3]. Notably, vitamin B12 deficiency has also been implicated in additional severe manifestations. These encompass various forms of dementia, underscoring the critical role of B12 in maintaining cognitive function. Furthermore, violent behavior and unexplained fatigue have been traced to B12 deficiency, highlighting its far-reaching impact on mental health, behavior, and overall well-being [4]. The neuropsychiatric consequences of vitamin B12 deficiency are well-documented, yet the underlying pathophysiological mechanisms remain poorly understood, with proposed explanations including alterations in one-carbon metabolism, disrupted folate metabolism, and genetic predisposition [5]. 

Reports of psychiatric patients without concurrent hematological symptoms are scarce. While isolated case studies suggest a link between vitamin B12 deficiency and acute psychosis, robust evidence is lacking [6]. This study presents eight patients presented to psychiatric facilities with Acute and Transient Psychotic Disorder (ATPD) and organic features, subsequently diagnosed with methylcobalamin deficiency without any typical neurological or hematological abnormalities. Notably, three cases originated from our prior published research. These findings contribute to the burgeoning evidence base underscoring vitamin B12 deficiency's role in precipitating acute psychotic episodes [7].

Vitamin B12 deficiency, a widespread condition, typically presents with hematologic and neuropsychiatric manifestations. However, psychiatric symptoms rarely precede anemia and seldom serve as the primary manifestation. This report documents eight exceptional cases where acute psychosis emerged without neurological symptoms or typical hematological abnormalities, prompting psychiatric facility utilizations.

## Materials and methods

This is a retrospective study showing the primary presentation of vitamin B12 deficiency as acute psychosis. Cases presented to the Psychiatric Department of Tata Main Hospital, Jamshedpur, India, who were diagnosed with ATPD as the primary diagnosis over the past five years from October 2019 to September 2024 were included initially in the study. A total of 225 patients diagnosed with ATPD were included in the study. Retrospective data evaluation of vitamin B12 was done. Twenty-four (10.6%) patients were found to have vitamin B12 deficiency. Of which, 11 (4.8%) were having other symptoms of vitamin B12 deficiency like paresthesia, dizziness, memory impairment, and jaundice and two patients were having adverse life events prior to the onset of symptoms. A total of 11 (4.8%) patients were found to have ATPD with vitamin B12 deficiency without any neurological or hematological symptoms of vitamin B12 deficiency and any past psychosocial stressors. Three patients had only visited twice and were lost to follow-up. The remaining eight (3.5%) patients were included in the study for further evaluation. Out of the eight patients, most had delusions and a few were having hallucinations and abnormal agitated behavior, and none of them had a family history of psychiatric disorders. The following selection criteria were used to identify the case for this study. Inclusion criteria include diagnosis of ATPD according to the International Classification of Diseases 10 (ICD 10) and vitamin B12 deficiency. Exclusion criteria include any other comorbid psychiatric illnesses and substance use other than nicotine. Hematological and neurological symptoms of vitamin B12 deficiency and significant life stressors prior to onset of illness.

## Results

Of the 225 ATPD patients, eight (3.5%) patients were shortlisted for the case series who had vitamin B12 deficiency without any hematological or neurological symptoms of vitamin B12 deficiency or any significant stressors. A detailed description of the cases is given in Table 1.

**Table 1:** Sociodemographic and clinical details of the eight selected cases

Variable	Case 1	Case 2	Case 3	Case 4	Case 5	Case 6	Case 7	Case 8
Age (Years)	31	54	34	16	67	20	34	27
Gender	Female	Male	Female	Male	Male	Male	Female	Male
Marital Status	Married	Married	Unmarried	Unmarried	Married	Unmarried	Married	Unmarried
Educational Level	Graduate	Graduate	Graduate	Higher Secondary	Graduate	Doing Graduation	Post Graduate	Post Graduate
Occupation	Unemployed	Skilled	Skilled	Student	Semi Skilled	Student	Skilled	Skilled
Residence	Urban	Urban	Urban	Urban	Urban	Urban	Urban	Urban
Food Habits	Vegetarian	Vegetarian	Vegetarian	Vegetarian	Vegetarian	Vegetarian	Vegetarian	Vegetarian
Religion	Hindu	Hindu	Hindu	Hindu	Hindu	Hindu	Hindu	Hindu
Comorbidity	No	Hyperthyroidism	No	No	Hypothyroidism	No	No	No
Family History of Psychiatric illness	No	No	No	No	No	No	No	No
Substance use	No	Nicotine	No	No	No	No	No	No

A detailed review of electronic medical records of all the eight patients was done. Case 2, despite being diagnosed with hyperthyroidism, his Thyroid Stimulating Hormone (TSH) was found to be normal as he was on regular medications for it. Additionally, Case 3 had an elevated bilirubin level, and a peripheral smear indicated dimorphic anaemia. Vitamin B12 injections and a blood transfusion of two units of packed red blood cells normalized her bilirubin and maintained on follow-up. Elevated indirect bilirubin suggested hemolysis associated with vitamin B12 deficiency, as her ultrasound abdomen and liver enzymes were normal [8]. Case 5 had an elevated TSH level (9.61 MiU/L) and a low hemoglobin (Hb) level (Table 2). There were no clinical symptoms of hypothyroidism in the patient. A systematic review and a meta-analysis done by Mohammed et al. [9] says that the median level of TSH for psychosis induced by hypothyroidism is 93 MiU/L with the presence of clinical symptoms of hypothyroidism, which ruled out the rare chance of hypothyroidism-induced psychosis in Case 5.

**Table 2:** Investigations Hb: Hemoglobin; MCV: Mean Corpuscular Volume; TSH: Thyroid Stimulating Hormone; UGIE: Upper Gastrointestinal Endoscopy; ALT: Alanine Transaminase; AST: Aspartate Transaminase; ALP: Alkaline Phosphatase Findings: All eight cases were found to be deficient in vitamin B12. Case 3 had low Hb for whom bone marrow examination was done and was found to have dimorphic anemia. MCV was higher for Cases 3, 4, 7 and 8.  TSH was higher for Case 5.

Investigation		Normal Range	Case 1	Case 2	Case 3	Case 4	Case 5	Case 6	Case 7	Case 8
Hb (gm/Dl)		11.5-16.5	12.0	11.7	5.7	14.7	11.2	13.9	12.3	14.4
MCV (fl)		76-96	87.6	85.5	115.3	98	94.6	79.8	101	101.3
TSH (Miu/L)		0.4-5.0	5.09	4.5	5.43	2.55	9.61	3.8	2.6	4.43
Vitamin B12 (ng/mL)		180-900	27.6	85	54	70	128	97	72	83
Folate (ng/mL)		2-20	7.18	5.83	5.55	8.53	5.87	4.83	6.74	5.36
Bone marrow		Not Applicable	Not done	Not done	Dimorphic Anemia	Not done	Not done	Not done	Not done	Not done
Neuro imaging		Not Applicable	Normal	Normal	Normal	Normal	Normal	Normal	Normal	Normal
UGIE		Not Applicable	Not done	Not done	Mild diffuse gastritis with normal gastric biopsy	Not done	Not done	Not done	Not done	Not done
Bilirubin (mg/dL)		0.2-1.2	1.1	0.44	2.19	0.56	1.05	0.85	0.76	0.78
Liver Enzymes (U/L)		ALT: 0-45 AST: 0-35 ALP: 53-141	Normal	Normal	Normal	Normal	Normal	Normal	Normal	Normal

Treatment history

All eight patients received comprehensive treatment, combining low-dose antipsychotics with vitamin B12 replacement therapy, as mentioned in Table 3. Antipsychotics were discontinued after one month in Cases 1 and 2 and after six months in Cases 3, 4, and 7. Doses were tapered to minimal effective levels in others. Regular vitamin B12 level monitoring at an interval of three months during the initial phase and six months thereafter ensured sustained improvement. Follow-up revealed asymptomatic patients, allowing either antipsychotic discontinuation or dose minimization over one year. Preventative measures included dietary modifications, vitamin B12 supplementation, and regular monitoring. These cases underscore vitamin B12 deficiency's potential to manifest exclusively as psychotic symptoms, emphasizing early recognition and treatment (Table 3).

**Table 3:** Treatment IM: Intramuscular; OP: Outpatient; IP: In Patient Cases 1, 5, 7, and 8 were treated with oral vitamin B12, while others were given IM 500 mcg vitamin B12. All cases were given low to intermediate doses of antipsychotics. Cases 2, 3, and 4 were managed on IP basis.

Treatment	CASE 1	CASE 2	CASE 3	CASE 4	CASE 5	CASE 6	CASE 7	CASE 8
Vitamin B12 replacement	Oral	IM	IM	IM	Oral	IM	Oral	Oral
Antipsychotic	Olanzapine 5 mg	Risperidone 2 mg	Risperidone 2 mg	Olanzapine 5 mg	Olanzapine 10 mg	Olanzapine 10 mg	Aripiprazole 2.5 mg	Risperidone 3 mg
Number of visits	4	3	6	3	3	9	8	4
Mode of care	OP	IP	IP	IP	OP	OP	OP	OP

## Discussion

Vitamin B12 is crucial for overall health, impacting multiple bodily systems. Its deficiency disproportionately affects the elderly, and the prevalence of neuropsychiatric disorders is 10-20% among Vitamin B12 deficient individuals. Common psychiatric manifestations include depression, mania, psychotic symptoms, cognitive impairment, and delirium. These symptoms underscore vitamin B12's vital role in maintaining mental well-being [10]. Mental disturbances caused by vitamin B12 deficiency have been documented as primary or sole symptoms, sometimes even appearing before serum cobalamin levels decrease [11].

Vitamin B12 deficiency-related psychosis, although rare, warrants attention due to its complex mechanisms. Research by Hutto suggests cobalamin and folate facilitate monoamine neurotransmitter synthesis in the brain through enhanced tetrahydrobiopterin (BH4) production [12]. Eight patients presented with ATPD symptoms, marked socio-occupational dysfunction and no history of psychiatric illness or substance abuse. Detailed interviews revealed no significant stressors contributing to their behavior, prompting exploration of organic etiologies.

Investigations uncovered significantly low serum vitamin B12 levels in all patients, likely attributed to vegetarian dietary habits lacking dairy products, meat, and eggs - primary sources of vitamin B12. Dietary analysis showed their daily intake consisted mainly of pulses, rice, and vegetables, with minimal dairy consumption. This heightened their risk due to inadequate vitamin B12 intake. Diagnosis typically relies on serum vitamin B12 measurement, but subclinical cases may exhibit normal levels. More sensitive screening methods involve assessing serum methylmalonic acid and homocysteine levels, which are elevated early in vitamin B12 deficiency. Given the patients' notably low vitamin B12 levels, further testing was unnecessary [13].

Vitamin B12 deficiency-induced acute psychosis is highlighted in eight rare cases in this study. Normal hematological parameters, specifically hemoglobin, hematocrit, and mean corpuscular volume (MCV), ruled out pernicious anemia in most of the cases. Cases 3, 7, and 8 had elevated MCV, but they were not anemic. Notably, significant stressors were absent in all the cases. Remarkable recovery was achieved with methylcobalamin and low-dose antipsychotics, with no symptom relapse post-antipsychotic discontinuation or dose reduction. A study conducted by Nicolas et al. replicated these findings, demonstrating that treatment with methylcobalamin yields similar outcomes in patients experiencing psychotic episodes [14]. These cases demonstrate vitamin B12 deficiency presenting as isolated acute psychosis, underscoring the importance of evaluating reversible causes. We recommend routine vitamin B12 level assessments in patients with mental and psychiatric disturbances to prevent severe, irreversible complications. Early detection and treatment are crucial. Dietary counselling and supplementation are vital.

Like other studies, our study also has limitations. Since the study was done on a limited sample of patients, our study cannot be generalized. We have no control for other confounding variables which would have contributed to the psychopathology. Like any other retrospective study, incomplete records and loss to follow-up are there in our study also. Since our study considers patients till remission, further long-term follow-up is needed to confirm vitamin B12 as the etiological factor for ATPD.

## Conclusions

Vitamin B12 deficiency-induced isolated acute psychosis, without hematological and neurological symptoms, is extremely rare. Despite its rarity, screening for vitamin B12 deficiency is crucial in patients with acute behavioral disturbances, particularly those following vegetarian diets. Early detection prevents irreversible psychiatric and neurological damage. Recommendations include routinely assessing vitamin B12 levels in patients presenting with first episode psychosis along with evaluation of hematological parameters and providing dietary counselling and supplementation.
